# Persistent Increase of Sympathetic Activity in Post-Acute COVID-19 of Paucisymptomatic Healthcare Workers

**DOI:** 10.3390/ijerph20010830

**Published:** 2023-01-01

**Authors:** Filippo Liviero, Maria Luisa Scapellato, Franco Folino, Angelo Moretto, Paola Mason, Sofia Pavanello

**Affiliations:** 1 Department of Cardiac, Thoracic, and Vascular Sciences and Public Health, University Hospital of Padua, Via Giustiniani 2, 35128 Padova, Italy; 2 University Hospital of Padova, Via Giustiniani 2, 35128 Padova, Italy

**Keywords:** SARS-CoV-2, paucisymptomatic COVID-19, HRV, cardiac autonomic dysfunction, increase of sympathetic activity, autonomic nervous system, TRPV-1, HCWs, health surveillance visit, general population

## Abstract

Healthcare workers (HCWs) represent a population with a significant burden of paucisymptomatic COVID-19, as the general population. We evaluated autonomic nervous system activity by means of heart rate variability (HRV) in HCWs during health surveillance visits. Short-term electrocardiogram (ECG) recordings were obtained 30 days (IQR 5.25–55.75) after a negative naso-pharyngeal swab for SARS-CoV-2 in 44 cases and compared with ECGs of 44 controls with similar age and sex distribution. Time and frequency domain HRV were evaluated. HCWs who used drugs, had comorbidities that affected HRV, or were hospitalized with severe COVID-19 were excluded. Frequency domain HRV analysis showed a significantly higher low/high-frequency power ratio (LF/HF) in the case study compared with controls (t = 2.84, *p* = 0.006). In time domain HRV analysis, mean standard deviation of normal-to-normal intervals (SDNN) and root mean square of successive RR interval differences (RMSSD) were significantly lower for cases compared with controls (t = −2.64, *p* = 0.01 and t = −3.27, *p* = 0.002, respectively). In the post-acute phase of infection, SARS-CoV-2 produces an autonomic imbalance mirrored by a reduction in HRV. These results are consistent with epidemiological data that suggest a higher risk of acute cardiovascular complications in the first 30 days after COVID-19 infection.

## 1. Introduction

Coronavirus disease 2019 (COVID-19) is the illness caused by severe acute respiratory syndrome coronavirus 2 (SARS-CoV-2) which has caused over 6 million deaths worldwide [1] since its first identification [2]. Healthcare workers (HCWs) belong to the occupational group with elevated risk of infection in general [3] and specially of SARS-CoV-2 infection [4]. Although the respiratory system is primarily affected by SARS-CoV-2, cardiac autonomic dysfunction is also emerging as a major issue in patients with previous SARS-CoV-2 infection [5,6]. Epidemiological data show that the risk of acute myocardial infarction was higher in the two weeks following COVID-19 (compared to controls), suggesting that the post-COVID-19 period involves an increased risk of acute cardiovascular complications [7]. Furthermore, in the first 30 days after infection, subjects with COVID-19 have an increased risk of death by incident cardiovascular disease spanning several categories, including heart failure and dysrhythmias [8]. In particular, a recent study conducted on 600,241 COVID-19-related deaths in the United States (reported between March 2020 and June 2021), showed that hypertensive diseases (19.6%), ischemic heart disease (10.9%), heart failure (7.7%), and cardiac arrhythmias (7.5%) were the most prevalent cardiovascular conditions amongst COVID-19-related deaths [9]. SARS-CoV-2 infection-induced stress is known to boost the sympathetic nervous system, leading to a neuro-hormonal implementation of pro-inflammatory cytokines with further development into sympathetic storming [10]. In our recent work, we hypothesized that the increase in cardiac events in SARS-CoV-2 survivors could be closely related to a potential autonomic imbalance of cardiac rhythm regulation, caused by TRPV-1 sensitization [11]. Thus, the characterization of cardiac autonomic function using heart rate variability (HRV) in infected patients is gaining interest, especially after recovery from COVID-19. Indeed, HRV is considered to be a sensitive and non-invasive method for the quantitative assessment of sympathetic and parasympathetic branches of the autonomic nervous system [12]. A higher HRV, modulated by efferent autonomic signals, is associated with a reduction of mortality in patients with cardiac diseases [13,14,15]. HRV measurements have been used in some case-control studies for the characterization of cardiac autonomic activity in severe hospitalized COVID-19 patients in the early phase of infection [16,17,18], after recovery from non-severe COVID-19 [19,20], and with long COVID-19 [21]. However, the results are conflicting since measurement methods, populations studied (inpatients, outpatients evaluated for specialist visits), and the severity and stage of infection at which testing was performed all differed between studies. In this work, we hypothesized that the presence of a persistent increase in sympathetic cardiac activity may account for epidemiological data that have detected an increased risk of post-COVID-19 cardiovascular events. Therefore, we evaluated autonomic cardiac balance and heart rate variability (HRV) parameters in HCWs during health surveillance visits that were carried out in the post-acute phase after recovery from paucisymptomatic or mild COVID-19 by means of this observational case-control study.

## 2. Materials and Methods

### 2.1. Study Population

A total of 96 unselected HCWs working in the University Hospital of Padua were screened with an HRV assessment. This was done during routine health surveillance carried out in compliance with legislative decree 81/08 and European Community Directive 90/679. Subjects included 48 HCWs in the post-acute phase of infection with a confirmed history of paucisymptomatic or mild COVID-19 infection between March 2021 and April 2022 and 48 control HCWs with similar age and sex distribution. From these subjects, eight HCWs were excluded because the collected electrocardiographic trace was not suitable for analysis (i.e., there were identification artefacts). The study population included 20 nightshift HCWs with a confirmed history of COVID-19 among the group of cases and 14 among the group of controls. HCWs who regularly work a full night shift at least 5 times a month were included as night workers. The “night period” started from 8 p.m. and ended 6 a.m. Since all HCWs were periodically screened with a SARS-CoV-2 naso-pharyngeal swab (NPS), as required by hospital protocol, we can reasonably exclude reinfections in the case group and asymptomatic COVID-19 infections in the control group. Subjects were excluded if they had an active COVID-19 infection, history of SARS-CoV-2 infection which required hospitalization or home oxygen treatment, or if they had severe respiratory or other major organ involvement. Subjects were also excluded if they were affected by or have a history of diabetes mellitus, cardiovascular diseases (i.e., moderate to severe valvular heart disease, coronary artery disease, arrhythmias), respiratory diseases (i.e., asthma, chronic obstructive pulmonary disease, sleep apnea), severe obesity, renal failure, thyroid disease, chronic liver disease and systemic inflammatory or autoimmune disorders, neurological disorders (i.e., cerebrovascular and Parkinson’s disease, Guillain-Barrè syndrome, polyneuropathy, multiple sclerosis), or malignancy. Moreover, subjects using drugs that could interfere with the analysis (i.e., beta-blockers, calcium channel blockers, inhaled or oral beta-mimetics, theophylline, or other drugs with potential chronotropic effects) were excluded. In the case group, 21 HCWs were fully vaccinated (with two or three doses) before the infection. In the control group, 40 HCWs were fully vaccinated (with two or three doses). All vaccinated subjects received the Pfizer-BioNTech mRNA vaccine (BNT162B2).

The study was approved by the local Research Ethics Committee (Protocol number = 267n/AO/22) and conducted in accordance with the ethical principles stated in the “Declaration of Helsinki”. All participants gave informed consent.

### 2.2. Assessment of Autonomic Cardiac Balance, HRV Parameters, and Blood Pressure

For the case group, short-term electrocardiogram (ECG) and blood pressure were recorded after a negative NPS for SARS-CoV-2 and after symptoms had disappeared (for at least three days). The median elapsed time from the negative NPS for SARS-CoV-2 to the short-term ECG recording was 30 days (IQR 5.25–55.75). Disease duration was defined as the period between the positive NPS (or the onset of symptoms if these were prior to the positive NPS) and the negative NPS for SARS-CoV-2. For controls, short-term ECG and blood pressure were recorded during periodical health checks. Subjects in both groups were instructed to avoid smoking, and to stop coffee and alcohol intake for 2 h and 48 h, respectively. They should have had sufficient (at least 8 h) rest and must not have worked the night shift before the test was performed. Blood pressure was measured once with a sphygmomanometer while the patient was lying calmly. HRV was assessed by an ECG performed in a supine position under physiologically stable conditions and using a device connected to the patient via two electrodes. HRV data were acquired by a Bluetooth acquisition system (BT16 Plus, FM, Monza, Italy). ECG was recorded for at least 5 min between 9 a.m. and 2 p.m., at rest and under ideal temperature conditions. HRV was analyzed using Kubios HRV software (ver. 3.3) [22]. Normal and aberrant complexes were identified and all adjacent intervals between normal beats over 5 min intervals were considered. We analyzed the spectral components (HRV frequency domain variables) as the absolute values of power (ms^2^) [12]. Power spectral density was analyzed with an autoregressive modeling-based method (AR spectrum), using the default value for the model order, i.e., 16. The main spectral components were very low frequency (VLF), low frequency (LF), high frequency (HF), and the LF/HF ratio. The area under the curve of the spectral peaks within the frequencies 0.01–0.4, 0.01–0.04, 0.04–0.15, and 0.15–0.40 Hz were defined as the total power (TP), very low-frequency power (VLF), low-frequency power (LF), and high-frequency power (HF), respectively. In order to normalize LF and HF, we used the total power within the frequency range of 0.01–0.4 Hz. The normalized low-frequency power (nLF = LF/TP) corresponds to an index of combined sympathetic and vagal modulation [23] as well as a baroreflex index [24,25], while the normalized HF power (nHF = HF/TP) represents an index of vagal activity. The low/high-frequency power ratio (LF/HF) is thus an index of sympathovagal balance. Time domain measures included the standard deviation of normal-to-normal RR intervals (SDNN), the root mean square of successive RR interval differences (RMSSD).

## 3. Sample Size Estimation

Sample size estimation for an unpaired t-test was applied to calculate the sample size. The calculation was computed through a STATA command by specifying a mean difference = 18 and standard deviation of differences = 28 for LF/HF as described in a similar study [16]. The group size to obtain statistical significance with α (two-tailed) = 0.05 and β = 0.80 was estimated to be *n* = 39 experimental subjects and 39 controls. The effect size was calculated for all HRV parameters, with the use of G*Power performing a post-hoc analysis after the completion of the study.

## 4. Statistical Analysis

Statistical analyses were performed with Minitab, LLC, version 18.0. The Kolmogorov–Smirnov test was performed to evaluate whether the variables were normally distributed. Continuous variables were presented as means ± SE or median (IQR 25–75) and categorical variables as frequency. Data with a wide dispersion were expressed in log transformed values. The Chi-square test with Yates correction was used for categorical variables where appropriate. For continuous data, a Student’s t-test was used for normally distributed variables. The Mann–Whitney U test was performed when indicated. Lastly, the influence of independent variables, including infection with SARS-CoV-2, age, sex, elapsed time from COVID-19 to ECG test, vaccination status, night work, body mass index, systolic blood pressure and palpitations on LF/HF, as dependent variable, was appraised by multiple linear regression analysis. Although the time–domain methods, especially the SDNN and RMSSD methods, can be used to investigate recordings of short durations, the frequency methods are usually able to provide more easily interpretable results in terms of physiological regulations. Therefore, we chose LF/HF as dependent variable in the multivariate analysis. All *p*-values less than 0.05 were considered significant.

## 5. Results

Characteristics of the study subjects are reported in Table 1.

**Figure 1:**
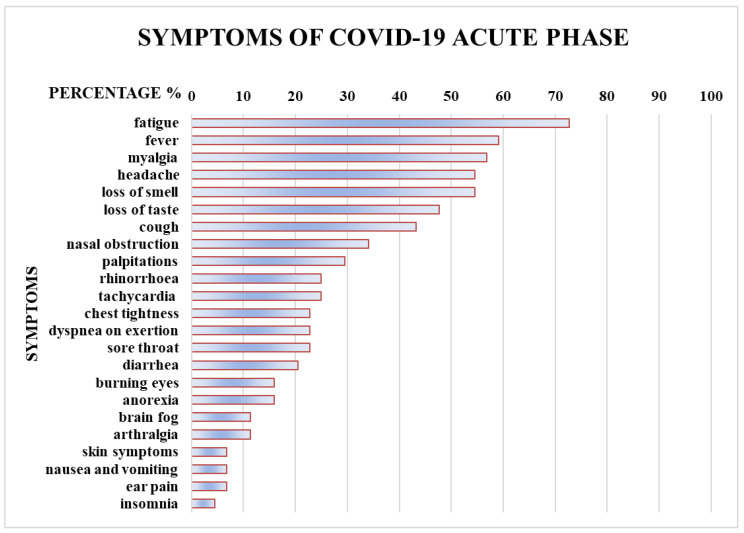
COVID-19 acute phase most commonly reported symptoms by HCWs at their health surveillance visits. Values are given as percentages (%).

No differences were found between the group of paucisymptomatic HCWs recovered from COVID-19 and the control group regarding age, gender, systolic and diastolic blood pressure, body mass index, and night shiftwork. Overall, 21 HCWs received a full vaccination cycle (with two or three doses) before SARS-CoV-2 infection, while 40 HCWs in the control group carried out the complete vaccination cycle (with two or three doses) (X^2^ (1, *n* = 88) = 17.3, *p* = 0.00032). In the case group, the median duration of the COVID-19 acute phase was 15.0 days (IQR 10.2–20.0). At their health surveillance visits, all subjects reported mild to moderate symptoms during the acute phase of the disease. The most commonly reported symptoms are listed in Figure 1. Cardiac symptoms (palpitations, tachycardia, and chest tightness) and systemic disautonomic symptoms (i.e., fatigue, headache, cough, dyspnea on exertion, brain fog) were well-represented (Figure 1).

Frequency domain analysis data was obtained, providing information on autonomic cardiac balance, as well as time domain analysis data providing information on HRV parameters. The data for recovered COVID-19 HCWs and the control group are shown in Table 2.

**Figure 2:**
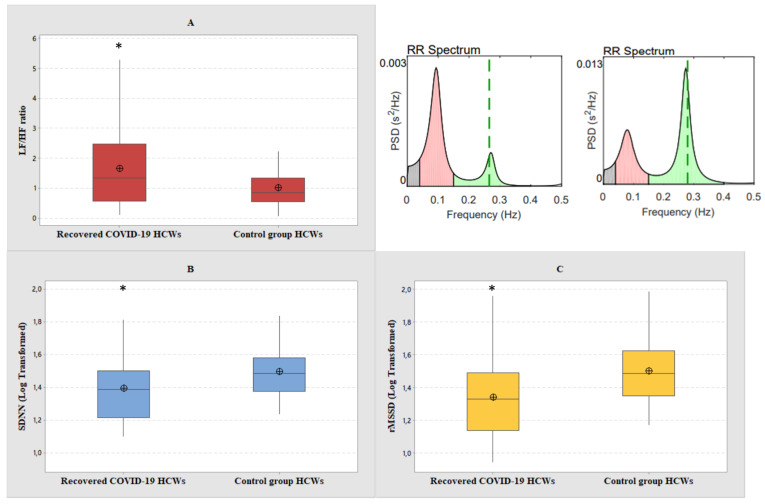
Boxplot graphical representation of (**A**) LF/HF ratio, (**B**) SDNN, log transformed values, and (**C**) RMSSD, log transformed values, among recovered COVID-19 and control group HCWs. In the upper right corner is reported the RR spectrum of a case (on the left) and its control (on the right). In box plots, the boundary of the box closest to zero indicates the 25th percentile, the line within the box marks the median, and the boundary of the box farthest from zero indicates the 75th percentile. The whiskers (error bars) above and below the box indicate the 95th and 5th percentiles. The circle with the inner cross indicates the mean value. * Student’s *t*-test, level of significance < 0.05.

Among the spectral components in the frequency domain HRV parameters, normalized high frequency power (nHF) was lower in the group of recovered COVID-19 HCWs compared with the group of control HCWs (t = −2.15, *p* = 0.03). Normalized low frequency power (nLF) was higher in the group of recovered COVID-19 HCWs compared with the group of control HCWs (t = 2.13, *p* = 0.03). Thus, the LF/HF ratio was higher in the group of recovered COVID-19 HCWs compared with the group of control HCWs (t = 2.84, *p* = 0.006) (Figure 2). Among the time domain parameters (Figure 2), both the mean SDNN and RMSSD were lower in the group of recovered COVID-19 HCWs compared with the control group (t = −2.64, *p* = 0.01 and t = −3.27, *p* = 0.002, respectively). Mean resting heart rate (HR) was in the range of normal values in both groups, although it was significantly higher in the group of recovered COVID-19 HCWs compared with the group of control HCWs (t = 2.42, *p* = 0.01). In our samples the following effect size were found for each HRV parameter, with a 95% confidence interval: 0.45 with a statistical power of 0.55 for nLF and nHF, 0.61 with a statistical power of 0.80 for LF/HF, 0.58 with a statistical power of 0.76 for SDNN, 0.70 with a statistical power of 0.90 for RMSSD, 0.51 with a statistical power of 0.65 for mean HR.

No significant correlations were found between cardiac parameters in the frequency and time domain (nLF, nHF, LF/HF, SDNN, RMSSD, and mean HR) and disease duration and elapsed time from COVID-19 to the ECG test (Supplementary Material, Tables S1 and S2). Subgroup analysis in recovered COVID-19 HCWs showed no significant differences between HRV parameters in the frequency and time domain (nLF, nHF, LF/HF, SDNN, RMSSD, and mean HR) in relation to sex, presence of cardiac symptoms and palpitations during the acute phase of infection, night shift work, and vaccination status (Supplementary Material, Tables S3–S6). Interestingly, unvaccinated recovered COVID-19 HCWs showed a trend to an increase in LF/HF (Mann–Whitney U-test, z = −1.26, *p* = 0.2) and a decrease in mean SDNN (Mann–Whitney U-test, z = −1.22, *p* = 0.22) and mean RMSSD (Mann–Whitney U-test, z = −1.89, *p* = 0.06) when compared to vaccinated recovered COVID-19 HCWs, although these differences were not statistically significant (Supplementary Material, Table S6). Multiple linear regression analysis showed that the principal determinants that increase LF/HF are confirmed to be age and infection with SARS-CoV-2 (Supplementary Material, Table S7).

## 6. Discussion

The main findings stemming from this study on paucisymptomatic COVID-19 HCWs evaluated in the post-acute phase of SARS-CoV-2 infection, when compared with control HCWs with similar age, sex, body mass index, blood pressure distribution, and comparable working conditions show: (1) an imbalance of autonomic cardiac regulation, characterized by a persistent increase in sympathetic activity, mirrored by an increase in nLF and LF/HF and decreased vagal activity, as shown by a reduction in nHF; (2) a reduction in HRV time domain parameters measured by mean SDNN and RMSSD. These results suggest that the persistent increase of sympathetic activity is associated with SARS-CoV-2 infection as well as the related inflammation and could be involved in post-COVID cardiovascular events. To our knowledge, this is the first study identifying a persistent increase of sympathetic drive and a reduction in HRV in paucisymptomatic COVID-19 HCWs in the post-acute phase, i.e., about days 30 after SARS-CoV-2 infection. Three previous studies analyzing sympathetic activity [16,17,18] gave results that were not entirely consistent. Two studies [17,18] found results consistent with ours in severe hospitalized patients in the early phase (a few days) of infection. They used 24-h Holter ECG [17] and beat-to-beat HRV analysis [18] to analyze autonomic function. Interestingly, they reported a higher LF/HF ratio in severe hospitalized patients than the level we detected in mild subjects. However, in the time domain parameters, Kaliyaperumal et al. [16] found an increase in parasympathetic tone and a decrease in the frequency domain measures (HF and LF) in hospitalized COVID-19 patients, which is in contrast with our results. Data from three other studies investigating cardiac autonomic function in the post-COVID period (more than 12 weeks after recovery) [19,20] and the long COVID syndrome [21] indicate that autonomic imbalance persists in the post-COVID period, as we found in the post-acute phase of infection. In particular, LF/HF values detected in patients with long COVID-19 syndrome [21] are comparable with our data, and were detected using the same short term ECG-HRV method as we used. One study [20], however, found a significant decreased in LF/HF in post-COVID patients with non-severe COVID-19, through 24-h Holter ECG. Interestingly, the LF/HF median value of the cases was similar to ours, but the control group was selected in a non-COVID-era among patients with palpitations (but no known autonomic imbalance, cardiovascular disease, or risk factors). Inappropriate control selection could be the reason for these unexplained results.

Viral infections are also known to be a trigger of dysautonomia [26]. Dysautonomia has been associated with neuroinflammation after infection, leading to a central dysregulation of the autonomic nervous system [26]. Data from human brain samples collected as part of routine autopsy procedures demonstrated that mild respiratory COVID-19 causes neuroinflammation and multi-lineage cellular dysregulation in the central nervous system [27]. It may be hypothesized that neuroinflammation in our paucysintomatic COVID-19 HCWs could be involved in the sympathetic activity increase we detected. Furthermore, our group recently studied the involvement of TRPV-1 in COVID-19 by modulating SARS-CoV-2 binding [11] and the consequent inflammatory conditions. The stimulation of TRPV-1 with the persistent increased sympathetic activity may therefore be suggested. TRPV-1 is, in fact, considered a “pathological receptor” that plays a key role in the transduction of noxious stimuli and in the preservation of inflammatory conditions [28]. TRPV-1 is involved in several inflammatory diseases, such as in inflammatory bowel disease (IBD), cutaneous neurogenic inflammation, brain inflammation, allergic asthma, cough, colitis, arthritis, hypersensitivity, chronic obstructive pulmonary disease (COPD), and autoimmune diseases [29,30,31]. Furthermore, activation of TRPV-1 increases the release of several pro-inflammatory molecules, for example neuropeptide substance P (sP) and cytokines such as interleukin 6 (IL-6), exactly the molecules that have been implicated in the pathophysiological events associated with COVID-19. Pro-inflammatory substances have been reported to be increased in COVID-19 cases and to reflect the severity of the illness [32]. Data in the literature show a general negative association between HRV and markers of inflammation [33] and cellular senescence [34], confirming the classical theory that the cholinergic anti-inflammatory pathway [35] acts as a link between the autonomic nervous system and the immune system. By revealing a suppression of parasympathetic activity and an increase in sympathetic drive in the post-acute phase of COVID-19 infection, our data may suggest that neuroinflammation could still be present at the time of analysis (in our study population). In fact, cardiac autonomic balance may be an indirect marker of post-COVID inflammation that might allow early identification of subjects with long COVID-19 that are at risk of clinical worsening [36]. Taken together, these data confirm that long-term follow-up of recovered COVID-19 HCWs, even those that were paucisymptomatic or had mild symptoms, is advisable to establish whether autonomic imbalance and lower HRV persist in this population.

Our work presents some limitations. The sample size was rather small, but the two groups of workers were comparable, so a lot of bias was excluded. Secondly, we did not include severe COVID-19 cases, which represented only 20% of the total [37]. We also did not measure inflammatory markers and thus the degree of inflammation at the time of the health surveillance visit; this measurement will be included in the follow-up of this study. Lastly, baseline exams (i.e., HR, blood pressure and short-term ECG) are not available because they were not provided in the study protocol which instead included a control group. Strengths of this work include the fact that HCWs represent an ideal study population, since they are less prone to selection bias compared with patients from a cardiology consult service who may have higher symptom burdens or more comorbidities than the general population. Moreover, HCWs are constantly under health surveillance, and the majority of infected subjects had milder symptoms/conditions with a better prognosis than hospitalized patients [38], which is what happens among the general population where the majority of cases are mild/moderate [39]. In our samples, the effect size for each main HRV parameter was greater than 0.50 with a considerable statistical power (i.e., about 0.80 for LF/HF, SDNN, and RMSSD). Furthermore, multiple linear regression analyses confirmed that the principal determinants that increase LF/HF are age and infection with SARS-COV-2. Thus, our results are robust because the main confounding factors (i.e., night shift work, concomitant pathologies, and use of drugs) were strictly controlled. Lastly, the follow-up model we have developed will be helpful for future assessment to better understand cardiac alterations in COVID-19 and its consequences for work capacity.

## 7. Conclusions

The most important findings can be summaries as follows.

SARS-CoV-2 is associated with an autonomic imbalance in the post-acute phase after recovery of paucysintomatic COVID-19.

The persistent increase of sympathetic activity reflected by a reduction in HRV may explain the epidemiological data on a higher risk of acute cardiovascular complications in the 30 days after SARS-CoV-2 infection.

Our results in HCWs, with a significant burden of paucisymptomatic COVID-19 as the general population, may have relevant public health consequences.

The simple, non-invasive, and inexpensive short-term HRV measurements can be used during health surveillance to help occupational physicians in issuing better judgments of fitness to work.

These measurements can also be used in the follow-ups for early identification of people (also within the general population) that may be at risk of clinical worsening, potentially developing into long-COVID syndrome.

Future research should certainly further test whether autonomic imbalance and lower HRV persist in the long-term, and whether they have a role in the development of post-COVID syndrome.

## Figures and Tables

**Table 1:** Characteristics of the study population.

Variables	Recovered COVID-19 HCWs	Control HCWs	*p*-Value
*N*, people	44	44	n.a.
Age, years	44.7 ± 9.98	45.2 ± 10.3	0.80
Male gender	12 (27.3%)	12 (27.3%)	n.d.
Systolic blood pressure, mmHg	131.7 ± 15.5	126.6 ± 11.2	0.08
Diastolic blood pressure, mmHg	82.9 ± 8.23	81.5 ± 6.70	0.35
Body mass index, kg/m^2^	24.5 ± 4.19	23.8 ± 3.98	0.47
Night shift workers	20 (45.4%)	14 (31.8%)	0.20 *
Vaccinated HCWs	21 (47.7%)	40 (90.9%)	0.00032 *
Disease duration, days	15.5 (10.2–20.0)	n.a.	n.a.
Duration from COVID-19 to ECG, days	30 (5.2–55.7)	n.a.	n.a.

Values are given as *n* and %, mean (± standard deviation) or median (IQR 25–75). *p*-values were calculated for continuous data with the Student’s *t*-test. * Chi-square test with Yates correction. n.a., not applicable. n.d., no difference.

**Table 2:** Frequency domain analysis for autonomic balance, and time domain analysis for HRV parameters (mean ± standard deviation and median IQR 25–75), among recovered COVID-19 and control group HCWs.

Variables	Recovered COVID-19 HCWs	Control Group HCWs	*p*-Value	Effect Size Value
nLF	53.6 ± 19.8	45.4 ± 16.2	0.03 *	0.45
nHF	46.4 ± 19.8	54.6 ± 16.2	0.03 *	0.45
LF/HF	1.66 ± 1.37	1.00 ± 0.66	0.006 *	0.61
SDNN ^a^	1.39 (1.21–1.50)	1.49 (1.37–1.58)	0.01 *	0.58
RMSSD ^a^	1.33 (1.14–1.49)	1.49 (1.35–1.62)	0.002 *	0.70
Mean HR, bpm	73.9 ± 8.67	69 ± 10.4	0.01 *	0.51

nLF, normalized low frequency; nHF, normalized high frequency; LF/HF, low/high-frequency ratio; SDNN, standard deviation of normal-to-normal R-R intervals; RMSSD, root mean square of successive RR interval differences; ^a^ Log transformed values. * Student’s *t*-test, level of significance < 0.05.

## Data Availability

Date were collected during routine health surveillance carried out in compliance with legislative decree 81/08 and European Community Directive 90/679. The data are not publicly available due to ethical and legal restrictions, as participants of this study did not agree for their data to be shared publicly.

## Supplementary Materials

The following are available online at https://www.mdpi.com/article/10.3390/ijerph20010830/s1. Table S1: Correlations between cardiac parameters and duration of the acute phase of the disease. Table S2: Correlations between cardiac parameters and elapsed time from COVID-19 to ECG test. Table S3. Subgroup analysis in recovered COVID-19 HCWs: cardiac parameters among males and females. Table S4: Subgroup analysis in recovered COVID-19 HCWs: cardiac parameters among subjects with and without cardiac symptoms and with and without palpitations during the acute phase of infection. Table S5: Subgroup analysis in recovered COVID-19 HCWs: cardiac parameters among night shift and daytime workers. Table S6: Subgroup analysis in recovered COVID-19 HCWs: cardiac parameters among vaccinated and non-vaccinated.
