# Supplementary material for: Persistent Increase of Sympathetic Activity in Post-Acute COVID-19 of Paucisymptomatic Healthcare Workers
Source: Int J Environ Res Public Health. 2023 Jan 1;20(1):830. doi: 10.3390/ijerph20010830 (PMC9820028; doi:10.3390/ijerph20010830)
Supplement: Supplementary file 1 [file ijerph-20-00830-s001.zip › ijerph-2048473-supplementary.pdf]

Supplementary Materials

Table S1. Correlations between cardiac parameters and duration of the disease's acute phase.

Variables	Pearson Correlation	*p*-Value
nLF	0.04	0.80
nHF	−0.04	0.80
LF/HF	−0.02	0.87
SDNN	−0.02	0.88
RMSSD	−0.03	0.84
Mean HR, bpm	0.01	0.43

Table S2. Correlations between cardiac parameters and elapsed time from COVID-19 to ECG-test.

Variables	Pearson Correlation	*p*-Value
nLF	0.07	0.64
nHF	−0.07	0.64
LF/HF	−0.08	0.61
SDNN	−0.17	0.25
RMSSD	−0.11	0.49
Mean HR, bpm	0.02	0.91

Table S3. Subgroup analysis in recovered COVID-19 HCWs: cardiac parameters among males and females.

Variables	Male, *n* = 12	Female, *n* = 32	*p*-Value
nLF	55.2 ± 16.6	53.0 ± 21.1	0.78
nHF	44.8 ± 16.6	47 ± 21.0	0.78
LF/HF	1.57 ± 1.04	1.69 ± 1.48	0.78
SDNN [a]	1.34 (1.21–1.56)	1.39 (1.22–1.50)	0.88
RMSSD [a]	1.33 (1.06–1.50)	1.33 (1.15–1.47)	0.98
Mean HR, bpm	74 ± 10.8	73.9 ± 7.93	1.00

Values are given as mean ± standard deviation and median (IQR 25–75). Mann-Whitney U-test was used between males vs females. [a] Log transformed values.

Table S4. Subgroup analysis in recovered COVID-19 HCWs: cardiac parameters among subjects with and without cardiac symptoms and with and without palpitations during the acute phase of infection.

Variables	Subjects with Cardiac Symptoms, *n* = 22	Subjects without Cardiac Symptoms, *n* = 22	*p*-Value
nLF	54.1 ± 19.8	53.0 ± 20.3	0.89
nHF	45.8 ± 19.8	47.0 ± 20.3	0.88
LF/HF	1.72 ± 1.48	1.60 ± 1.28	0.87
SDNN [a]	1.34 (1.21–1.47)	1.42 (1.24–1.58)	0.23
RMSSD [a]	1.34 (1.09–1.44)	1.32 (1.15–1.53)	0.50
Mean HR, bpm	73.0 ± 6.97	74.7 ± 10.2	0.58
**Variables**	**Subjects with Palpitations, *n* = 13**	**Subjects without Palpitations, *n* = 31**	***p*-Value**
nLF	51.5 ± 20.6	54.5 ±19.8	0.63
nHF	48.4 ± 20.6	45.5 ± 19.8	0.64
LF/HF	1.49 ± 1.16	1.73 ± 1.46	0.64
SDNN [a]	1.42 (1.22–1.52)	1.35 (1.21–1.49)	0.64
RMSSD [a]	1.40 (1.15–1.55)	1.29 (1.14–1.49)	0.47
Mean HR, bpm	74.4 ± 7.33	73.7 ± 9.27	0.78

Values are given as mean ± standard deviation and median (IQR 25–75). Mann-Whitney U-test was used between subjects with cardiac symptoms vs subjects without cardiac symptoms during the acute phase of infection. [a] Log transformed values.

Table S5. Subgroup analysis in recovered COVID-19 HCWs: cardiac parameters among night shift and workers.

Variables	Night Shift Workers, *n* = 20	Daytime Workers, *n* = 24	*p*-Value
nLF	53.2 ± 18.7	53.9 ± 21.1	0.90
nHF	46.7 ± 18.7	46.0 ± 21.0	0.90
LF/HF	1.58 ± 1.27	1.72 ± 1.47	0.90
SDNN [a]	1.43 (1.29–1.52)	1.31 (1.17–1.48)	0.19
RMSSD [a]	1.35 (1.21–1.47)	1.27 (1.08–1.49)	0.35
Mean HR, bpm	74.9 ± 9.27	73.0 ± 8.24	0.76

Values are given as mean ± standard deviation and median (IQR 25–75). Mann-Whitney U-test was used between night shift vs daytime workers. [a] Log transformed values.

Table S6. Subgroup analysis in recovered COVID-19 HCWs: cardiac parameters among vaccinated and non-vaccinated.

Variables	Vaccinated, *n* = 21	Non-Vaccinated, *n* = 23	*p*-Value
nLF	49.5 ± 19.9	57.3 ± 19.4	0.21
nHF	50.4 ± 19.9	42.7 ± 19.4	0.21
LF/HF	1.37 ± 1.07	1.92 ± 1.56	0.20
SDNN [a]	1.42 (1.22–1.56)	1.34 (1.19–1.47)	0.22
RMSSD [a]	1.39 (1.19–1.55)	1.26 (1.08–1.38)	0.06
Mean HR, bpm	72.7 ± 6.87	75.0 ± 10.0	0.40

Values are given as mean ± standard deviation and median (IQR 25–75). Mann-Whitney U-test was used between vaccinated vs non-vaccinated. [a] Log transformed values.

Table S7. Multiple regression analysis (model a) of the influence of SARS-COV-2 infection, age, sex, elapsed time from COVID-19 to ECG test, vaccination status, night work, body mass index, systolic blood pressure and palpitations on LF/HF.

Variables	B	R	t	*p*-Value
SARS-COV-2 infection	0.72	0.24	2.15	**0.03**
Age	0.03	0.27	2.51	**0.01**
Sex	0.05	0.02	0.17	0.87
Elapsed time from COVID-19 to ECG test	−0.003	−0.06	−0.52	0.61
Vaccination status	−0.14	−0.15	−1.38	0.17
Night work	0.05	0.02	0.19	0.84
Body mass index	−0.004	−0.01	−0.11	0.91
Systolic blood pressure	0.007	0.08	0.76	0.45
Palpitations	−0.33	−0.11	−0.94	0.34
